# Network Analysis of Cross-Correlations on Forex Market during Crises. Globalisation on Forex Market

**DOI:** 10.3390/e23030352

**Published:** 2021-03-15

**Authors:** Janusz Miśkiewicz

**Affiliations:** 1 Institute of Theoretical Physics, University of Wrocław, 50-204 Wrocław, Poland; janusz.miskiewicz@uwr.edu.pl; 2 Physics and Biophysics Department, Wrocław University of Environmental and Life Sciences, 50-375 Wrocław, Poland; janusz.miskiewicz@upwr.edu.pl

**Keywords:** time series analysis, cross-correlations, power law classification scheme, network analysis, globalisation, entropy

## Abstract

Within the paper, the problem of globalisation during financial crises is analysed. The research is based on the Forex exchange rates. In the analysis, the power law classification scheme (PLCS) is used. The study shows that during crises cross-correlations increase resulting in significant growth of cliques, and also the ranks of nodes on the converging time series network are growing. This suggests that the crises expose the globalisation processes, which can be verified by the proposed analysis.

## 1. Introduction

The economy is a human activity where interactions are particularly important. The mutual impacts are caused by an exchange of goods, services, and co-operation, but also competition, company overtaking, industrial espionage, etc. In result, one can observe a grouping among entities in the form of co-operation, branches, common interest, or competition on the market. These effects are the subject of many research fields, e.g., portfolio analysis [1,2,3,4], market structure analysis [5,6], globalisation researches [7,8,9], and many others.

The main tool for exploring the nature of interdependence among entities (companies, branches, shares, countries, etc.) is the cross-correlation analysis. In fact, this term is gathering a great variety of methods. Just mentioning the most often used: classical variance analysis and Pearson correlation coefficient [10,11,12,13,14,15], cointegration analysis [16,17,18,19], multifractal analysis [20,21,22,23], random matrix theory [24,25,26,27], power law classification scheme [28,29,30], or entropy-based methods [31,32].

The range of problems investigated by cross-correlation analysis is very broad, starting from sociology, economy, econophysics [20,23,33,34], transport [35,36], genome analysis, biology, food network, biochemistry network, science collaboration network [37], up to sport [38], and many others.

Within this study, the globalisation is analysed by the power law classification scheme (PLCS). In difference to other cross-correlation methods, such as detrended fluctuation analysis (DFA) [39,40,41,42,43,44,45] or the Pearson coefficient-based method [12,15,46,47], which are focused on noise correlation, PLCS is focused on trends. In the case of globalisation, trends seem to be more important, because they reflect similarities in evolution rather than mutual dependence and sensitivity to external impulses. Besides that, PLCS analysis allows for observing different features—medium-range correlations. On the other hand, the method is sensitive to long-term deterministic correlations that are related to “fundamental” effects [48]. The research analyses the currency exchange rate time series as an objective measure of mutual relationship and interactions among economies. The currency exchange rates are one of the most important parameters of the economy status. There are several platforms where the exchange of currencies occur. The best known and one of the most important from the global point of view is the Forex market, which is focused on the institutional market. Besides that, there are many other exchange platforms that are aimed at individuals, such as exchange office, banks, and Internet exchange systems. The present study focuses on the Forex exchange time series, since the main goal is the analysis of economy globalisation, particularly cluster formation during stock market crises.

## 2. Methods

The power law classification scheme (PLCS) is focused on correlations of trends [28]. The algorithm will be shortly described here for the clarity of presentation and convenience of the reader.

Let assume that there are two time series recorded simultaneously with the same length *N*. In the first step, the subseries from the initial point *k* are taken and the Manhattan distance between them calculated. The procedure is repeated for each k∈{1,…,N}. At this point, the series of cumulative Manhattan distance is obtained. Each point of this series corresponds to a different “*k*”. Finally, the power law function is fitted to the cumulative Manhattan distance series. The power of the fitted function diminished by one defines the correlation strength.

### Example of Application

Let us assume that there are two time series that are generated by the linear functions:

$$f_{1}\left(t\right)=a_{1}\cdot t,\;\;\;f_{2}\left(t\right)=a_{2}\cdot t.$$

The data are registered in equal intervals e.g., t=1,2,…,N. The generated time series are denoted as $f_{1}$ and $f_{2}$. Subsequently, the cumulative series of the Manhattan distance between series $f_{1}$ and $f_{2}$ is equal to

$$MD\left(k\right)=\sum_{i=1}^{k}|a_{1}-a_{2}\left|i=\right|a_{1}-a_{2}|\frac{(1+k)k}{2},$$

so

$$MD\left(k\right)=\frac{|a_{1}-a_{2}|}{2}\left(k+k^{2}\right).$$

The last step is the fitting of the power law function. The most popular method is fitting the linear function to the log-log transformed data e.g., (ln(k),ln(MD(k))). Of course, the quality of the fit depends on the series length. In the case of the analysed functions $f_{1}$ and $f_{2}$, the fitted exponent for the first 100 data points is equal to 1.922, but, for 1000 data points, is equal to 1.982 and asymptotically approach 2. The observed uncertainity is the result of numerical limitations of the computer memory while calculating the logarithm. In order to obtain the correlation strength, one has to diminish the exponent of the fitted function by one and finally obtains 1. Of course, this result is in agreement with the linear relationship between the considered functions. Other examples and more detailed analysis can be found in [28,29,30].

The results of PLCS analysis can be classified into two categories:

α<0  when the correlation strength is smaller than zero—the distance between time series is decreasing, the time series are converging.
α>0  when the correlation strength is greater than zero—the distance between time series is increasing, and the time series are diverging.

The special case of α=0 is observed when the time series are overlapping [28].

In the present study, the *time evolution* of correlation strength is analysed; therefore, the additional correlation window parameter is introduced $T_{c}$. The correlation strength is calculated in a moving time window, so the appropriate subseries of the length $T_{c}$ are taken and the correlation strength between them calculated; subsequently, the starting point is shifted by one day and the procedure is repeated.

The application of PLCS to a time series gives symmetrical correlation matrix with $\frac{N^{2}-N}{2}$ unique elements (*N*—is the number of time series elements considered). Therefore, to conclude, it should be further analysed. The popular strategy is to construct a network, e.g., Minimum Spanning Tree or others. However, PLCS allows for distinguishing two types of cross-correlation: convergent and divergent time series. Therefore, in this paper, the following two networks are constructed:

- converging time series networks, i.e., only the nodes (representing the currency time series) with a correlation strength smaller than one are connected, and
- diverging time series network, i.e., only the nodes with a correlation strength greater than one are connected.

Clearly, the first type of network is focused on the time series approaching each other, while the second on the time series increasing differences.

In the presented study, the grouping of currencies was analysed, particularly the clique and community formation were investigated. Therefore, the following network features were calculated: the clique size evolution, the community number, the frequency of the connection on the graph, the evolution of the network node rank distribution, and the rank node entropy.

*Clique size* evolution is obtained by calculating the size of the biggest clique for each of the generated networks. The clique size evolution illustrates a process of unification of the market. Indeed, if the giant clique is observed, then one type of correlation is dominating on the market and, on the contrary, if the size of the biggest cluster is small, then the correlation matrix consists of a variety of correlation type.
*Community number* is obtained by measuring the number of community structure partitions that group nodes, such that there is a higher density of edges within the community than between them. This parameter is weaker than the clique number, but still allows observing grouping on Forex market.
*The frequency of connection* on the graph is the measure where the frequency of being connected on the graph is analysed. The most important feature of this measure is the ability to distinguish the most stable connections in the considered period.
*Node rank distribution* is the analysis where the most detailed information regarding the graph is obtained. The rank of nodes is an important feature allowing for observing the hierarchy of a network and is often used to determine network type [49,50,51]. This measure gives very detailed information regarding the graph. It may be considered as a quick overview of the network main features, e.g., if it is densely connected or whether each node is only connected with a small number of links.
*Rank node entropy* is the Shannon entropy that is defined in the standard way (Equation (1)), where the evolution of the entropy of node rank is calculated.
  (1) $$S=\sum_{i}-p_{i}\ln p_{i},$$
  where $p_{i}$ is the probability of i-th rank. A summation is done over all ranks of nodes present in the network.

Those analyses are performed for both types of networks (diverging and converging).

## 3. Data

### 3.1. Data Source

The foreign exchange market (Forex) is a global network of brokers and computers that serves as a place of currency exchange. The market is active from Monday morning in Asia to Friday afternoon in New York and is active 24 h per day.

The most important feature of the Forex market (and very natural) is that the exchange is quoted in pairs in difference to stock markets, where each stokes has its value. It is important to mention that the arbitrage on Forex is possible in a short time scale [52,53,54]. This induces some bias on the analysis, because the choice of the base currency may influence the results, particularly on the very short time scale. On the other hand, one can distinguish a group of leading currencies, which are the most frequently traded: US dollar, euro, and Japanese yen, which are dominating in the market. The bias resulting from the arbitrage is reduced by PLCS feature—due to the averaging procedure. Moreover, in the present study, the euro, as the leading currency, has been chosen as a central currency and exchange rate time series investigated in this paper.

Within this study, the daily exchange rates registered on the Forex market were analysed. The data set consists of 34 time series with the euro as the base currency. The following exchange rates have been investigated: AR, CZK, AUD, DKK, BGN, EGP, BRL, HKD, CAD, HRK, CHF, HUF, IDR, CNY, ISK, JPY, KRW, MXN, MYR, NAD, NOK, NZD, PHP, PLN, RON, RUB, SEK, SGD, THB, TRY, TWD, UAH, USD, and ZAR. Standard abbreviations are used. The period is from 03.09.1996 until 05.02.2020, i.e., 1000 data points.

Within the considered period, one can distinguish several crises (on a regional and global scale). The crises are playing a special role in the presented analysis, because we can expect highlighting the globalisation processes. To mention the most serious crises within the considered interval: 1997—Asian financial crisis [55], 1998—Russian crisis [56], 1999—Argentine crisis [57], early 2000s recession [58], dot-com bubble [59], 2008 financial crisis [60], 2010 European sovereign debt crisis [61], national government debt-crises (Spanish, Greek, Russian, and Turkish), and others. Those crises are discussed in view of the performed analysis results.

### 3.2. Descriptive Statistics of the Series

The exchange rate time series were converted into return time series by Equation (2).

(2) $$r_{i}\left(t\right)=\frac{a_{i}\left(t\right)-a_{i}\left(t-1\right)}{a_{i}\left(t-1\right)}$$

where $a_{i}$ denotes the analysed time series.

Table 1 presents the statistical properties of the investigated time series. The mean value of the exchange rate returns of the considered time series was in the interval $\left(-0.629\times 10^{-4},9.087\times 10^{-4}\right)$, so the average daily fluctuations are rather small, and they are close to zero. However, the range of observed returns is significant—the lowest noticed return was −0.282, while the greatest was 0.585. The next considered parameter—standard deviation—is particularly important, because it is broadly used as a measure of volatility. When comparing the values of standard deviation and the mean, one can notice that the dispersion is huge. The standard deviation is two orders of magnitude greater than the mean. Another important piece of information is given by skewness analysis. Many of the time series have skewness that is much different from zero, which means that the return distribution is asymmetric. The lowest skewness is observed for CHF exchange rate return, while the highest value is achieved for EGP. The last discussed statistical feature is the result of kurtosis, which is much bigger than one and are leptokurtic for all considered time series. The highest values are observed for EGP, CHF, AR, UAH, IDR, and RUB.

**Figure 1:**
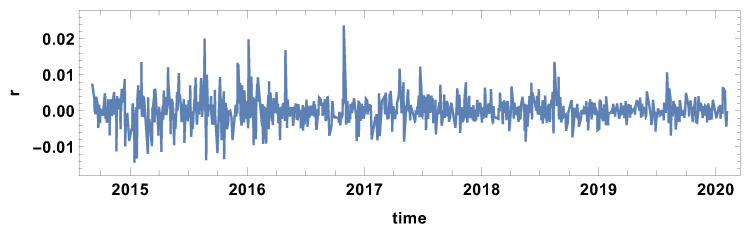
The mean value of the exchange rates return of the considered time series.

Additionally, the time evolution of the mean return exchange rate is presented in Figure 1. This graph allows for obtaining a general idea of Forex market evolution, particularly to distinguish the periods of instability of the market.

## 4. Results

The moving time window technique must be used to study the time evolution of cross-correlation. The results of the analysis depend on the correlation time window length. The long time window smooths the fluctuations and it can hide important system features. On the other hand, the short time window does not provide a good quality fit of the power law, and the fluctuations are more apparent in the analysis. Therefore, PLCS algorithm was applied for three time window lengths: $T_{c}\in \left(20,60,120\right)$, which correspond to a month, quarter, and half of the year period.

### 4.1. Month Time Window

**Figure 2:**
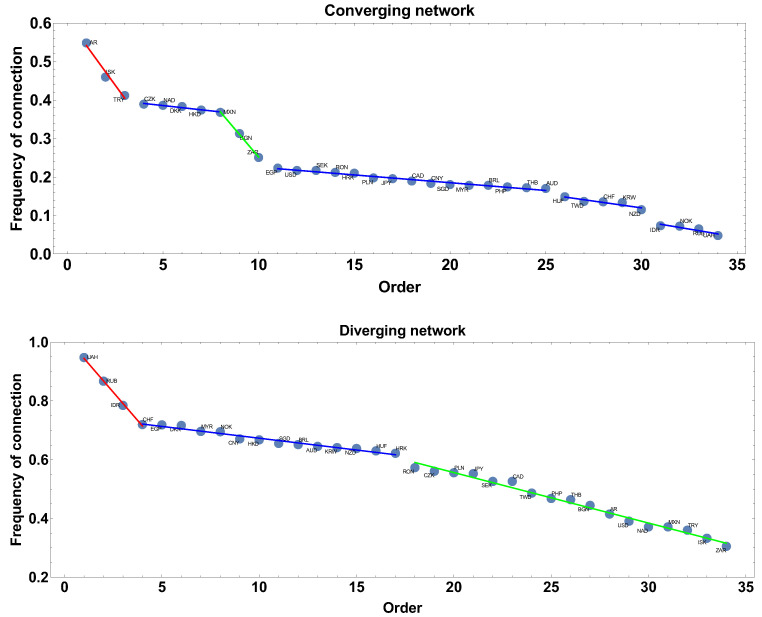
The frequency of connection presented in descending order. The time window $T_{c}=20$ days. The blue line denotes a group of currencies of similar frequency of being connected on the network.

The frequency of connection is the first parameter investigated here. This parameter informs how often the correlation strength was converging or diverging, so how stable was the correlation in the analysed period. In the case of the diverging correlation strength network, the result is presented in Figure 2.

Applying the linear fit to the frequency rank allowed for distinguishing three groups of currencies. The first group is marked by the red line: UAH, RUB, and IDR. The second group is marked by the blue line: CHF, EGP, DKK, MYR, NOK, CNY, HKD, SGD, BRL, AUD, KRW, NZD, and HUF. The third group is marked by the green line: RON, CZK, PLN, JPY, SEK, CAD, TWD, PHP, THB, BGN, AR, USD, NAD, MXN, TRY, ISK, and ZAR.

In the case of the network construction based on the converging time series, i.e., the correlation strength α<0 the frequency of connection ranks are presented in Figure 2 and denoted as the converging network. In this case, six groups can be distinguished. Taking more detailed analysis into account, the following groups can be pointed out: the first, marked by the red line AR, ISK, and TRY, and the second, denoted by the blue line, consists of CZK, NAD, DKK, HKD, and MXN. The third group, marked by the green line consists of two members BGN, ZAR. The fourth group is the biggest EGP, USD, SEK, RON, HRK, PLN, JPY, CAD, CNY, SGD, MYR, BRL, PHP, THB, and AUD. The two other groups are formed by HUF, TWD, CHF, KRW, NZD and IDR, NOK, RUB, UAH. Although both graphs are, in some sense, complementary, divergent correlation graphs are constructed under the condition that on the graph there are currencies with α>0, while the divergent graph under condition α<0 the graphs in Figure 2 are not simple mirror images of each other. This is because, in the analysis, the whole correlation matrix is investigated and a given currency may be present on both types of graphs at the same time (it might be convergent with respect to one time series and divergent with concerning another). Particularly interesting are the groups denoted by the blue lines. These groups consist of currencies with similar frequency of being present in the network (divergent or convergent respectively), so the method introduces a natural categorization of time series.

**Figure 3:**
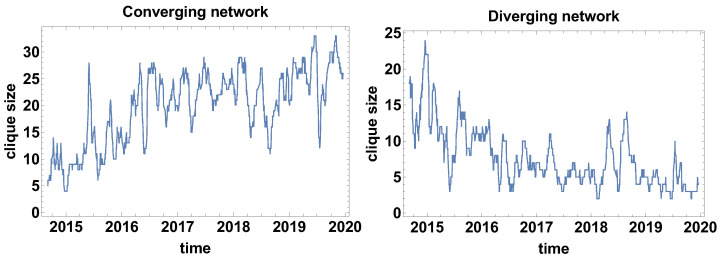
The biggest clique size evolution. Time window size $T_{c}=20$ days.

Clique size evolution. In the context of correlation strength network, the cliques are special formations. The cliques are the fully connected group of currencies, with the same type of correlations. Figure 3 presents the clique size evolution graphs for both types of networks. The main advantage of the clique size evolution analysis is the possibility to observe the clique formation in time. The converging time series network that is presented in Figure 3 shows that the biggest clusters were formed in the fourth quarter in 2014, which can be interpreted as the moment when most of the time series were converging, so the differences were decreasing. The clique was formed by 24 currencies. At the other maxima, the formed clusters were not so large and they were in the interval 17–10 currencies. The local maxima were observed in mid-2015, the second and third quarter of 2016, the first quarter of 2017, the second and third quarter of 2018, and the second quarter of 2019. It is also worth noticing that the average level of clique size before 2017 was on the level of 12 currencies, whereas, afterwards, the average value becomes about five time series. Thus, the significant decrease of the clique size is noticeable.

The changes in the average size of the clique that are observed for the converging time series graph are supported by the analysis of the clique size evolution for the diverging time series graph Figure 3. In this case, the initial average size of the clusters was increased from the size of about 10 currencies to more than 23 currency time series. In the high frequency (short time window) analysis, the clique size in the diverging time series network is of high variance, which means that there is no stable tendency. The clusters are formed temporarily. However, the significant value of the cluster size suggests that the majority of the time series are divergent.

**Figure 4:**
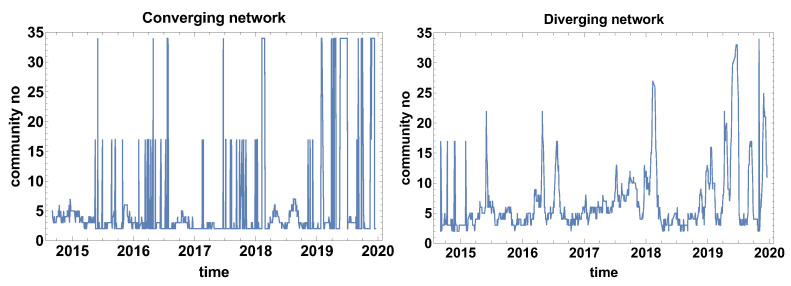
Evolution of the communities number. Time window size $T_{c}=20$ days.

The structure analysis of the network was continued by calculating the number of communities that formed on the network. This structure community analysis is based on a weaker constraint than the clique search. Another difference to the biggest clique size is the number of communities is analysed instead of the biggest clique size. The number of communities algorithm looks for the subgraph group with nodes with a higher density of connections than the other part of the network (indifference to the clique that is a fully connected subgraph). Figure 4 presents the results of the number of community analysis.

Figure 4 presents the evolution of the community number that is observed for both types of networks. Intriguing is the evolution of community number in the case of the converging network (the correlation strength α>0). Three levels of community number can be distinguished in Figure 4 for the converging network these are the ground level where a few communities are observed and two other states of 17 and 34 nodes. Such a big number of communities suggests that they are of very small size (one or at most a few nodes), additionally, the huge increment denotes that shift of the time window by one day has changed the situation significantly. This can be interpreted as either the period is extremely unstable or the correlation strength is approximately close to zero and small changes of the data set have affected the classification of the time series. This observation suggests that, in future applications of the method, it might be worth considering the introduction of an additional class of time series cross-correlation α≈0. Besides the two-state period, the other local maxima are not spectacular, because they are not exceeding seven communities.

The graph presenting the evolution of the community size for the diverging network (Figure 4) differs significantly from the converging network. In this case, except for the initial part at the end of 2014, the two-level behaviour is not observed. Therefore, the diverging network seems to be more robust to the network switching effect. Similarly to the converging network, the “baseline” of the community number can be distinguished (2–5 communities). Several clear maximums can be distinguished in the case of the diverging network quantity of community evolution: June 2015, April and July 2016, February 2018, and several maxima in 2019. 2019 was the most unstable year out of those analysed when many times the network was split into a big number of small communities.

The evolution of the community number for the converging network might suggest that the time window size $T_{c}$ is too short and fluctuations significantly influence the results of the analysis.

**Figure 5:**
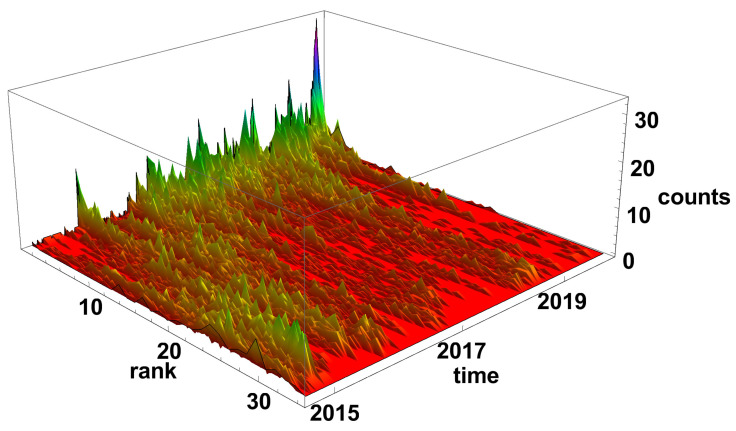
Evolution of the rank nodes histogram for converging network. The time window size $T_{c}=20$ days. The counts denote how many times the node of given rank (number of links) was observed on the network.

**Figure 6:**
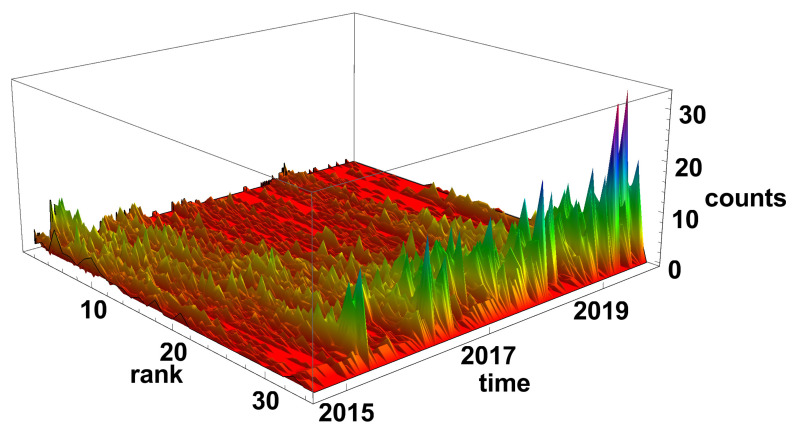
Evolution of the rank nodes histogram for diverging network. The time window size $T_{c}=20$ days. Counts denotes how many times the node of given rank (number of links) was observed on the network.

Figure 5 and Figure 6 present the evolution of the node rank histogram for converging and diverging time series networks, respectively. When analysing the evolution of the node rank histogram for the converging time series network shown in Figure 5, it can be observed that in 2015 and 2016 the nodes with a significant number of links (*k* > 20) are dominating. Whereas, in 2017 and later, the nodes with the low number of links (*k* < 15) are dominating. A short exception is observed in 2018 (during the Chinese crisis) when nodes with a high number of links were clearly present in the network. In 2019 and later, the nodes with a small number of links are prevailing on the converging network.

The evolution of the diverging time series network histograms is presented in Figure 6. Initially, in 2015, the nodes with a small number of links are most evident, but, since 2016, the situation has changed and the nodes with a high number of connections are the most common on the network. It is particularly well seen at the end of 2019 and the beginning of 2020, when nodes with the degree k>30 are dominating on the network.

**Figure 7:**
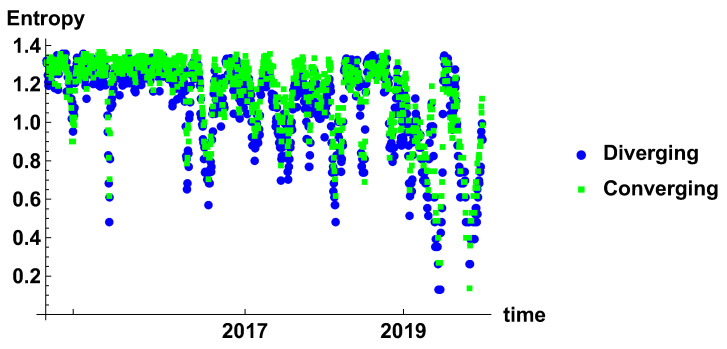
Evolution of the rank node entropy for diverging and converging networks. The time window size $T_{c}=20$ days. The blue circles and green squares denote the entropy of diverging and convergent network, respectively.

Figure 7 presents the evolution of rank node entropy. There are no significant differences between the generated networks. Particularly interesting are the minima, which correspond to the situation where there is a significant group of nodes of the same rank. Although several minima can be distinguished, they do not form a clear evolution; this is due to the noise influence. This results indicate that the time window is rather too short to obtain a clear evolution of the system.

### 4.2. Quarter Time Window

**Figure 8:**
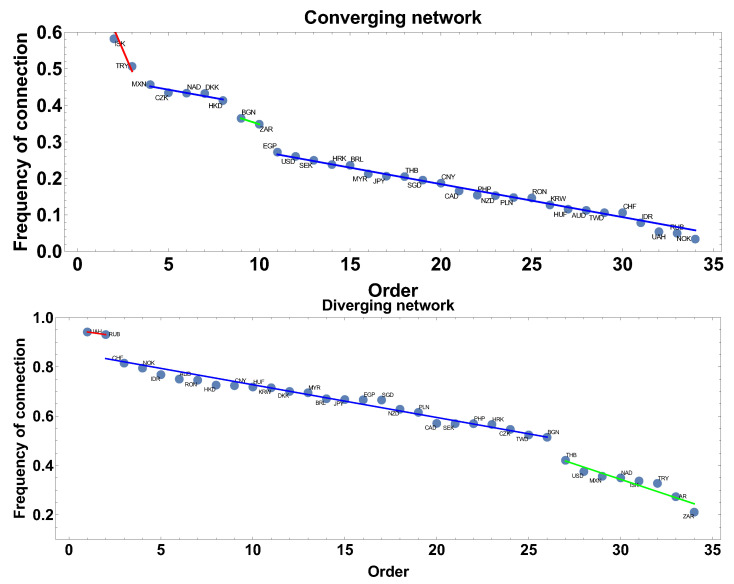
The frequency of connection presented in descending order. The time window $T_{c}=60$ days. The blue line denotes group of currencies of similar frequency of being connected on the network.

Extending the size of the time window $T_{c}$ to 60 days results in filtering high frequency changes, which were observed in the one-month time window. Following the same scheme of network feature analysis shown in Section 4.1, the discussion starts from the frequency of being connected. The results are presented in Figure 8. In the case of networks constructed with the constraint of the converging time series, the most frequent connections are ISK and TRY, while, for the divergent time series network, the most frequent observed currencies are UAH and RUB, which are present in 94% and 93% of the constructed network. The blue line denotes the group of currencies with similar frequency of the network member. For the converging time series network, the biggest group has a frequency in the interval 27–3%, being rather low, while, in the second type of network considered here, the frequency is in the interval 82–51%, so the probability of connection is significantly higher.

**Figure 9:**
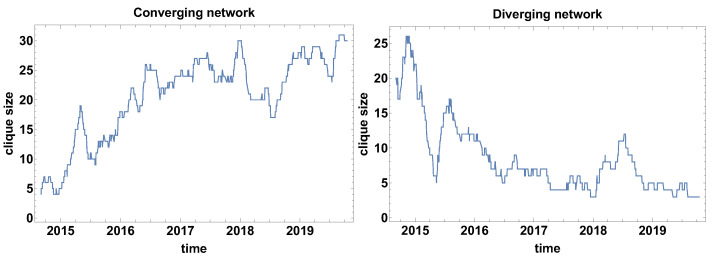
The biggest clique size evolution. Time window size $T_{c}=60$ days.

Figure 9 shows the time evolution of the biggest clique size (so the clusters of a fully connected set of currencies). It can be noticed that the divergent and convergent time series networks results are on average complementary—the size of the cliques in convergent time series is growing in time, but in divergent time series are decreasing. Of course, the graphs differ in details. Moreover, the general similarity does not apply to the position and magnitude of extreme points. For the converging network, as in Figure 9, six local extremes can be distinguished. The local maxima are observed in April 2015, March 2016, May–June 2016, April–June 2017, January 2018 (which is the highest maxima of 30 nodes in one clique), and the local minimum in June 2018. The clique size evolution in the diverging time series network has approximately four local extremes. The first maximum is observed at the end of 2014, which is followed by a very deep minimum in April 2015. The decrease of the clique size is enormous, because, at the first maximum, there are 26 nodes in the clique, while at the minimum the biggest clique consists of 5 nodes, so the biggest clique size decreased by 21 nodes. Immediately after that minimum, the biggest clique is growing to achieve the size of 17 nodes in August 2015. Subsequently, the clique size is relatively slowly decreasing to the level of 4–7 nodes. The last maximum is observed in July 2018.

**Figure 10:**
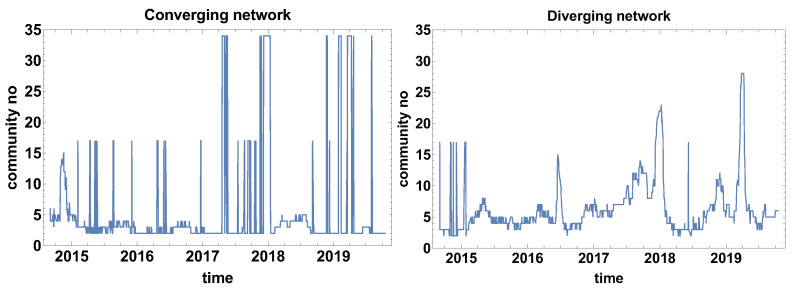
Evolution of the community number. The time window $T_{c}=60$ days.

Figure 10 presents the evolution of the community number. In the evolution of community number of converging networks, one can distinguish three levels: the ground state, where the community approximately 3–6 communities, the second level of 16–17 communities, and the third level of 34 communities. Because the border between converging and diverging time series is α=0, the bistable behaviour of the graph means that a significant group of currencies is at the border and a small shift of the time window position is changing their classification. A similar observation was made for the evolution of community number for $T_{c}=20$ days. As it was already mentioned, the additional class of α≈0 is not introduced here due to the clarity of the analysis, because the main aim of the study is to verify the properties of the algorithm. The additional class should be considered in such a case in, e.g., commercial analysis.

The bistable behaviour of the size of the community size is also observed in the diverging network shown in Figure 10 at the end of 2014. Afterwards, the bistable evolution is not observed and several clear maxima can be noticed: June 2016, at the end of 2017, and in April–May 2019. It can be observed that, due to the longer time window, the number of maxima has been reduced when compared to the previously discussed case, as in Figure 4.

**Figure 11:**
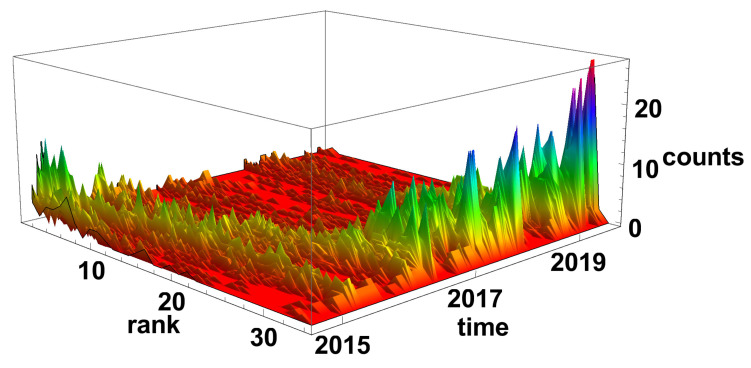
Evolution of the rank nodes histogram for converging network. The time window size $T_{c}=60$ days. Counts denote how many times the node of given rank (number of links) was observed on the network.

**Figure 12:**
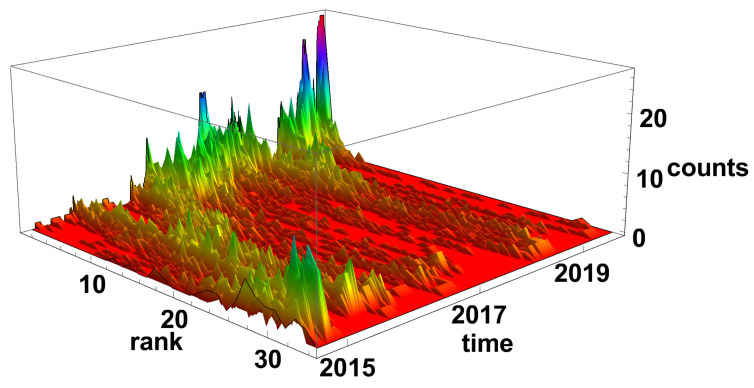
Evolution of the rank nodes histogram for diverging network. The time window size $T_{c}=60$ days. Counts denote how many times the node of given rank (number of links) was observed on the network.

The evolution of the node rank histograms for converging and diverging networks are presented in Figure 11 and Figure 12, respectively. In both types of network, two periods can be distinguished: the most common is the high-rank nodes or the reverse situation—the low-rank nodes. The converging time series network, as in Figure 11, is, in general, complementary to the diverging network case, as in Figure 12. At the end of 2014, the low-rank nodes are dominating, while, in 2016, 2017, and 2019, the high-rank nodes are prevailing in the histograms. Combining the results of the rank node histograms evolution with the clique size analysis, where huge clusters are observed, as in Figure 9, it can be concluded that the generated networks are very close to a fully connected network.

In the case of the diverging time series network, as in Figure 12, the nodes of high rank are observed at the end of 2014, at the end of 2015, and the beginning of 2016. A very special situation occurs at the beginning of 2015, when there is no dominating group of nodes, but all the ranks of nodes are present in the histogram. In 2017, at the end of 2018, and then the beginning of 2019, the networks are divided into small subgraphs. In the mid of 2018, the increase of high-order nodes is observed—this situation can be related to the Chinese crisis.

**Figure 13:**
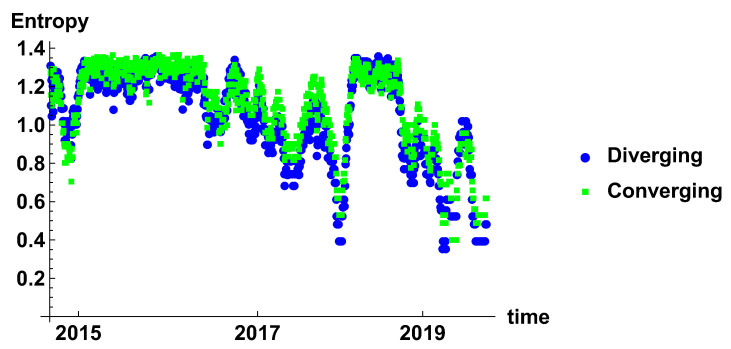
Evolution of the rank node entropy for diverging and converging networks. The time window size $T_{c}=60$ days. The blue circles and green squares denote the entropy of diverging and convergent network respectively.

Figure 13 presents the entropy of the rank node distribution for the time window of $T_{c}=20$ days. In this case, the influence of noise is significantly reduced. The different periods can be clearly distinguished. Initially, in 2015 the decrease of entropy is observed, which is the effect of domination of high rank nodes in the histograms. The period of stable high entropy follows, which lasts until the mid of 2016. Later, oscillation appears, which are combined with the decrease of the minimum value to achieve minimum in the beginning of 2018. In 2018, another period of maximum entropy is observed. It seems that level 1.4 is the maximum entropy observed in these networks and can be considered as a measure of the stability of the market. A significant lowering of the entropy may be considered as a signature of the crisis.

### 4.3. Half Year Time Window

**Figure 14:**
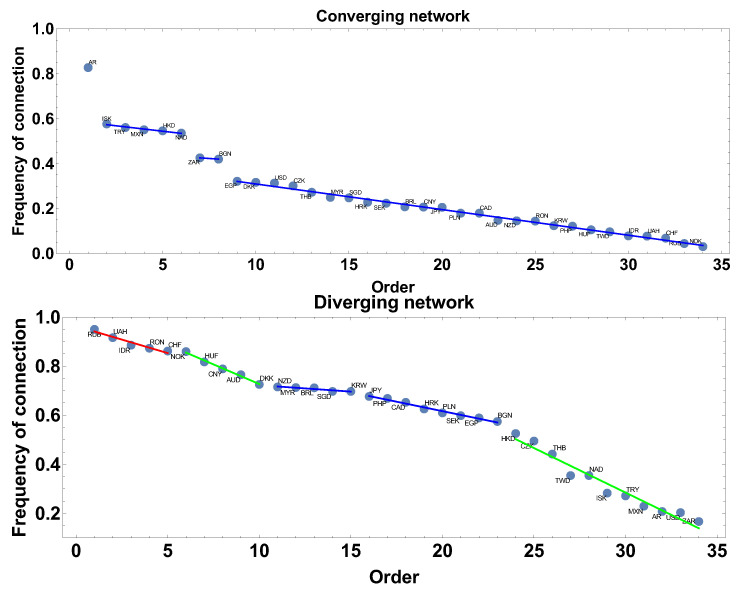
The frequency of connection presented in descending order. The time window $T_{c}=120$ days. The blue line denotes group of currencies of similar frequency of being connected on the network.

This subsection contains the results obtained for the longest time window $T_{c}=120$ days. Figure 14 presents the results of the frequency of connection of nodes to the network for both types of networks. In the case of the converging network, AR is the most frequent currency, which is present in 83% generated graphs. This node is separated and does not belong to any group. The first group, which can be distinguished in this analysis, consists of five currencies: ISK, TRY, MXN, HKD, and NAD. Currencies of this group are connected to others in 58–54% of networks. The second group consists of two currencies: ZAR and BGN. The last group is the biggest one—26 currencies. Within this group, the frequency of being connected is rather low: from 32% to 3%.

The frequency of being connected on a divergent time series graph is slightly different because only two groups of similar frequency, i.e., without significant differences between consecutive elements, can be distinguished. The first group consists of five currencies: NZD, MYR, BRL, SGD, and KRW, and their frequency is varying from 72% to 70%. This group is followed by the second one: JPY, PHP, CAD, HRK, PLN, SEK, EGP, and BGN with the frequencies from 68% to 57%.

**Figure 15:**
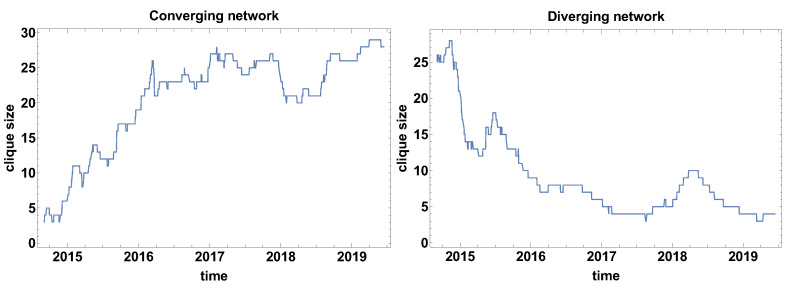
The biggest clique size evolution. Time window size $T_{c}=120$ days.

Figure 15 presents the biggest clique size evolution for the time window $T_{c}=120$ days. When comparing to the previously discussed cases, i.e., $T_{c}=20,60$ days, the smoothing effect of the time window size is clearly visible. In this case, the biggest clique size for the converging time series network is asymptotically increasing with the exception in the middle of 2018, which can be related to the Chinese stock market crisis. An analogous maximum is observed in the graph presenting the biggest clique size evolution for the diverging network shown in Figure 15.

**Figure 16:**
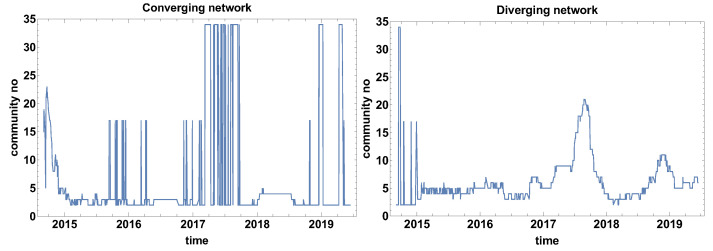
Evolution of the community number. The time window $T_{c}=120$ days.

Figure 16 presents the evolution of the community number on the graph for the time window $T_{c}=120$ days. In the case of the converging network, the observed previously switching effect between two states for shorter time windows is also present in this case. However, in difference to the previous analyses, there is a period when the network brakes into separate nodes. This is the second and third quarter of 2017. At this time, in the community number of the diverging network graph, the maximum is reaching the value of 20 nodes. Simultaneously, the high number of communities is observed in diverging and converging networks this suggests that no clear tendency (or significant correlation) is present in the market. This finding agreed with the fact that, at this time, there was no serious global crisis.

**Figure 17:**
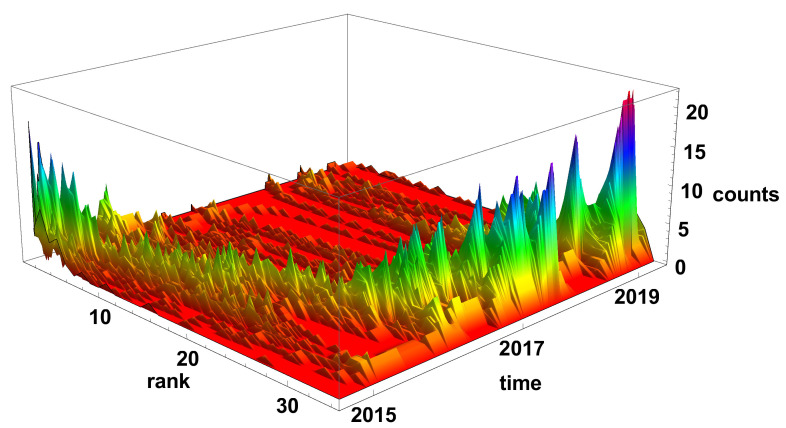
Evolution of the rank nodes histogram for converging network. The time window size $T_{c}=120$ days. Counts denotes how many times the node of given rank (number of links) was observed on the network.

**Figure 18:**
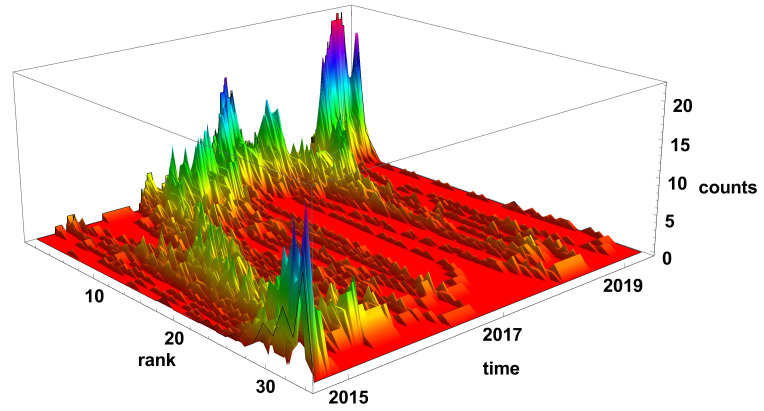
Evolution of the rank nodes histogram for diverging network. The time window size $T_{c}=120$ days. Counts denote how many times the node of given rank (number of links) was observed on the network.

The node rank histogram evolution that were obtained for the time window $T_{c}=120$ days are presented in Figure 17 and Figure 18. In both graphs, the change node rank structure is clearly visible. In the case of converging time series network, as in Figure 17, at the beginning of the analysed period, i.e., at the end of 2014 and in the first quarter of 2015 the low-rank nodes are prevailing in the network, while, from 2016, the high-rank nodes are dominating. Differently from the already analysed rank histograms evolutions for shorter time windows ($T_{c}=20$ and $T_{c}=60$ days) in the case of $T_{c}=120$ days, the process of network transition from domination of low-rank nodes to high-rank nodes, prevailing network is a kind of continuous process. The transformation process lasts approximately a year when the nodes are gaining connections. The significant shift of the maximum position of the low-rank nodes is observed in mid-2018, probably due to the Chinese stock market crisis.

The diverging network rank node histogram evolution, as shown in Figure 18, is complementary to the converging series network. At the end of 2014, the high-rank nodes are prevailing in the histogram. During 2016, the node rank frequency of occurrence is evolving from high node rank domination to low-rank nodes prevailing in 2016. Finally, since 2016, the low-rank nodes have dominated the network except for mid-2018.

**Figure 19:**
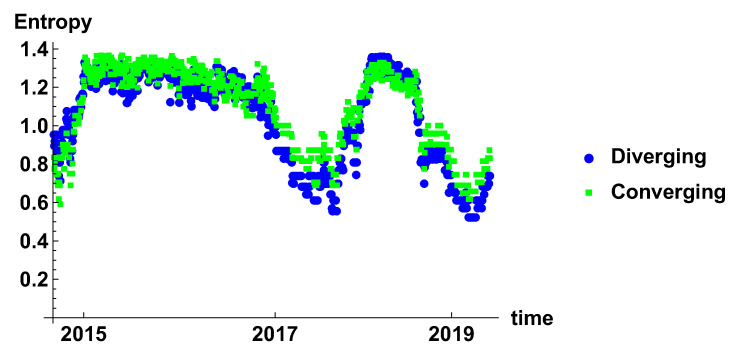
Evolution of the rank node entropy for diverging and converging networks. The time window size $T_{c}=120$ days. The blue circles and green squares denote the entropy of diverging and convergent network respectively.

The rank node entropy evolution that is observed in the case of the time window $T_{c}=120$ days is presented in Figure 19. The long time window results in significant filtering of the time series. In this case, the most stable effects can be observed. In the presented results, there are two such events—one in 2017 and the second in 2019. The outcomes of the analysis for the half-year time window confirm the previous observations that the crisis is characterised by a low value of the entropy of the rank node distribution.

## 5. Conclusions

The presented study investigates the cross-correlations among currency exchange rates on Forex market by the PLCS algorithm, followed by network analysis. The PLCS method is focused on the trend correlations and unlike other methods, allows to observe cross-correlation of trends. The results of this paper show that crises influence trend correlations. The convergent and divergent networks are not simple mirrors of each other. Because the network is constructed with the cross-correlation matrix, the introduced constraint may reveal a different feature, e.g., the community number observed in the converging network presents a two-state evolution that is rarely observed in a diverging network. Particularly interesting is the biggest cluster size analysis, which is sensitive to crisis occurrence. Particularly, the change of the cluster size can expose the severity crisis. The third feature investigated here is the frequency of the connection, which verifies the stability of the connection. Currencies are forming groups concerning the frequency of connections to the network. It might give an opportunity to develop a new classification of currencies with respect to their relationship to the group. The last performed analysis—the rank node histogram evolution—provides the most detailed information about the structure and evolution of the cross-correlation among currencies. The analysis of the rank node entropy is particularly interesting. The obtained results suggest that entropy might be a synthetic measure of crisis. Of course, this conclusion needs further analysis, but the presented results are very promising.

A very special outcome of this analysis is that, in recent times, e.g., 2017, the structure of the observed networks has changed and depending on the type of the network (converging or diverging) the high or low-rank nodes are prevailing. It means that the cross-correlation in the Forex market has changed significantly. The observed changes in the biggest clique size and the number of communities are the results of globalisation, which are more transparent during crises. In this special condition, correlations and mutual dependence are exposed. Of course, the results depend on the choice of central currency and the analysis can be repeated for other central currencies. However, the main aim of this paper was establishing new analysis methods, so the detailed analysis of the role of the central currency choice is left for other studies. The additional results are the analysis of the role of the time window length. The presented results allow for estimating the window size with the requested quality of research. It is not recommended to use time windows shorter than 20 days. Of course, extending the size of the time window improves the quality of the results from the statistical point of view, and it filters the high frequency changes exposing the long-term proprieties. Although this aspect was not discussed here, longer time windows might be more appropriate for forecasting.

## Figures and Tables

**Table 1:** Statistical properties of the exchange rate returns.

Currency	Mean	Median	Std	Max	Min	Skewness	Kurtosis
	$\cdot 10^{-4}$	$\cdot 10^{-4}$	$\cdot 10^{-2}$				
AR	9.087	3.836	1.413	0.403	−0.126	11.147	261.7
CZK	−0.471	−0.740	0.486	0.093	−0.064	1.291	41.6
AUD	0.324	−2.245	0.760	0.079	−0.050	0.743	10.8
DKK	0.053	0	0.048	0.079	−0.009	−0.560	84.1
BGN	1.393	0.452	0.845	0.063	−0.060	0.324	6.4
EGP	3.513	0.665	1.261	0.586	−0.075	21.336	961.4
BRL	3.334	−0.718	1.182	0.129	−0.1108	0.513	15.8
HKD	−0.079	0	0.663	0.055	−0.070	−0.094	8.4
CAD	−0.140	−1.480	0.674	0.044	−0.043	0.201	5.7
HRK	0.351	0.135	0.492	0.049	−0.053	0.092	18.2
CHF	−0.629	0	0.468	0.088	−0.159	−6.186	304.3
HUF	1.335	0.289	0.595	0.070	−0.062	1.174	20.1
IDR	4.849	0	1.802	0.462	−0.207	5.287	134.0
CNY	−0.313	0.329	0.834	0.050	−0.062	−0.102	8.4
ISK	1.392	−0.991	0.876	0.145	−0.133	1.199	71.2
JPY	0.061	2.313	0.849	0.083	−0.116	−0.606	17.0
KRW	1.050	−1.545	1.084	0.158	−0.232	−0.678	78.1
MXN	1.944	0	0.904	0.068	−0.091	0.221	10.1
MYR	1.033	−0.253	0.773	0.068	−0.070	0.129	13.0
NAD	2.782	−0.375	1.100	0.184	−0.101	1.500	25.9
NOK	0.585	−0.937	0.530	0.050	−0.082	−0.350	23.5
NZD	0.162	−3.306	0.783	0.057	−0.051	0.341	6.3
PHP	1.367	1.125	0.799	0.111	−0.130	−0.039	29.5
PLN	0.615	−1.442	0.642	0.057	−0.048	0.609	9.6
RON	5.452	0.675	0.908	0.192	−0.096	3.521	76.2
RUB	6.074	1.393	1.651	0.347	−0.282	4.050	124.6
SEK	0.570	−0.450	0.475	0.036	−0.039	0.228	8.4
SGD	−0.159	0	0.595	0.043	−0.052	−0.154	7.0
THB	0.412	0.367	1.016	0.171	−0.067	1.045	25.0
TRY	9.005	4.949	1.177	0.267	−0.086	4.395	89.3
TWD	0.09	−0.232	0.654	0.068	−0.069	0.079	9.6
UAH	6.469	0	1.732	0.554	−0.215	8.250	258.0
USD	−0.082	0	0.672	0.077	−0.077	−0.046	11.6
ZAR	2.768	−1.856	1.113	0.121	−0.143	0.259	18.1

## References

[1] Chandra P. (2017). Investment Analysis and Portfolio Management.

[2] Briston R.J. (2017). The Stock Exchange and Investment Analysis.

[3] Yu J.N. (2020). Research on Financial Portfolio Analysis in the New Era. J. Phys. Conf. Ser..

[4] Safitri I.N.N., Sudradjat S., Lesmana E. (2020). Stock portfolio analysis using Markowitz model. Int. J. Quant. Res. Model..

[5] Auer R.A., Schoenle R.S. (2016). Market structure and exchange rate pass-through. J. Int. Econ..

[6] Corbet S., Lucey B., Urquhart A., Yarovaya L. (2019). Cryptocurrencies as a financial asset: A systematic analysis. Int. Rev. Financ. Anal..

[7] Levitt T. (1993). The globalization of markets. Readings in International Business: A Decision Approach.

[8] Beck U. (2018). What Is Globalization?.

[9] Scholte J.A. (2005). Globalization: A Critical Introduction.

[10] Wang G.J., Xie C., Stanley H.E. (2018). Correlation structure and evolution of world stock markets: Evidence from Pearson and partial correlation-based networks. Comput. Econ..

[11] Piao L., Fu Z. (2016). Quantifying distinct associations on different temporal scales: Comparison of DCCA and Pearson methods. Sci. Rep..

[12] Mantegna R.N. (1999). Hierarchical structure in financial markets. Eur. Phys. J. B.

[13] Miśkiewicz J., Ausloos M. (2008). Correlation measure to detect time series distances, whence economy globalization. Phys. A Stat. Mech. Its Appl..

[14] Miśkiewicz J. (2011). Distance matrix method for network structure analysis. Statistical Tools for Finance and Insurance.

[15] Mantegna R.N., Stanley H.E. (1999). Introduction to Econophysics: Correlations and Complexity in Finance.

[16] Granger C.W. (1988). Causality, cointegration, and control. J. Econ. Dyn. Control.

[17] Johansen S. (1988). Statistical analysis of cointegration vectors. J. Econ. Dyn. Control.

[18] Watson M.W. (1994). Vector autoregressions and cointegration. Handb. Econom..

[19] Adebola S.S., Gil-Alana L.A., Madigu G. (2019). Gold prices and the cryptocurrencies: Evidence of convergence and cointegration. Phys. A Stat. Mech. Its Appl..

[20] Wang J., Shang P., Ge W. (2012). Multifractal cross-correlation analysis based on statistical moments. Fractals.

[21] El Alaoui M., Bouri E., Roubaud D. (2019). Bitcoin price–volume: A multifractal cross-correlation approach. Financ. Res. Lett..

[22] Oświęcimka P., Drożdż S., Forczek M., Jadach S., Kwapień J. (2014). Detrended cross-correlation analysis consistently extended to multifractality. Phys. Rev. E.

[23] Pal M., Rao P.M., Manimaran P. (2014). Multifractal detrended cross-correlation analysis on gold, crude oil and foreign exchange rate time series. Phys. A Stat. Mech. Its Appl..

[24] Ren F., Zhou W.X. (2014). Dynamic Evolution of Cross-Correlations in the Chinese Stock Market. PLoS ONE.

[25] Utsugi A., Ino K., Oshikawa M. (2004). Random matrix theory analysis of cross correlations in financial markets. Phys. Rev. E.

[26] Plerou V., Gopikrishnan P., Rosenow B., Amaral L.A.N., Guhr T., Stanley H.E. (2002). Random matrix approach to cross correlations in financial data. Phys. Rev. E.

[27] Pharasi H.K., Sharma K., Chakraborti A., Seligman T.H. (2019). Complex market dynamics in the light of random matrix theory. New Perspectives and Challenges in Econophysics and Sociophysics.

[28] Miśkiewicz J. (2013). Power law classification scheme of time series correlations. On the example of G20 group. Phys. A Stat. Mech. Its Appl..

[29] Miśkiewicz J. (2016). Cross-correlations of the Forex market using power law classification scheme picture. Acta Phys. Pol. A.

[30] Miśkiewicz J., Tadla A., Trela Z. (2019). Does the monetary policy influenced cross-correlations on the main world stocks markets? Power Law Classification Scheme analysis. Phys. A Stat. Mech. Its Appl..

[31] Miśkiewicz J. (2020). Entropy of Globalizing World Macroeconomy Time Series Analysis. Acta Phys. Pol. A.

[32] Teng Y., Shang P. (2017). Transfer entropy coefficient: Quantifying level of information flow between financial time series. Phys. A Stat. Mech. Its Appl..

[33] Ramchand L., Susmel R. (1998). Volatility and cross correlation across major stock markets. J. Empir. Financ..

[34] Kristoufek L. (2014). Detrending moving-average cross-correlation coefficient: Measuring cross-correlations between non-stationary series. Phys. A Stat. Mech. Its Appl..

[35] Jin S.T., Kong H., Sui D.Z. (2019). Uber, public transit, and urban transportation equity: A case study in new york city. Prof. Geogr..

[36] Zaarane A., Slimani I., Hamdoun A., Atouf I. (2019). Real-Time Vehicle Detection Using Cross-Correlation and 2D-DWT for Feature Extraction. J. Electr. Comput. Eng..

[37] Hellsten I., Lambiotte R., Scharnhorst A., Ausloos M. (2007). Self-citations, co-authorships and keywords: A new approach to scientists’ field mobility?. Scientometrics.

[38] Ausloos M. (2020). Rank–size law, financial inequality indices and gain concentrations by cyclist teams. The case of a multiple stage bicycle race, like Tour de France. Phys. A Stat. Mech. Its Appl..

[39] Chen Z., Ivanov P.C., Hu K., Stanley H.E. (2002). Effect of nonstationarities on detrended fluctuation analysis. Phys. Rev. E.

[40] Hu K., Ivanov P.C., Chen Z., Carpena P., Eugene Stanley H. (2001). Effect of trends on detrended fluctuation analysis. Phys. Rev. E.

[41] Fan Q., Liu S., Wang K. (2019). Multiscale multifractal detrended fluctuation analysis of multivariate time series. Phys. A Stat. Mech. Its Appl..

[42] Kwapień J., Drożdż S. (2012). Physical approach to complex systems. Phys. Rep..

[43] Bryce R.M., Sprague K.B. (2012). Revisiting detrended fluctuation analysis. Sci. Rep..

[44] Höll M., Kiyono K., Kantz H. (2019). Theoretical foundation of detrending methods for fluctuation analysis such as detrended fluctuation analysis and detrending moving average. Phys. Rev. E.

[45] Oświęcimka P., Kwapień J., Drożdż S. (2006). Wavelet versus detrended fluctuation analysis of multifractal structures. Phys. Rev. E.

[46] Mantegna R.N., Palágyi Z., Stanley H.E. (1999). Applications of statistical mechanics to finance. Phys. A Stat. Mech. Its Appl..

[47] Bonanno G., Lillo F., Mantegna R. (2001). High-frequency cross-correlation in a set of stocks. Quant. Financ..

[48] Bouchaud J.P., Cont R. (1998). A Langevin approach to stock market fluctuations and crashes. Eur. Phys. J. B.

[49] Hassan M.K., Islam L., Haque S.A. (2017). Degree distribution, rank-size distribution, and leadership persistence in mediation-driven attachment networks. Phys. A Stat. Mech. Its Appl..

[50] Bauer B., Jordán F., Podani J. (2010). Node centrality indices in food webs: Rank orders versus distributions. Ecol. Complex..

[51] Hou B., Yao Y., Liao D. (2012). Identifying all-around nodes for spreading dynamics in complex networks. Phys. A Stat. Mech. Its Appl..

[52] Gębarowski R., Oświęcimka P., Wątorek M., Drożdż S. (2019). Detecting correlations and triangular arbitrage opportunities in the Forex by means of multifractal detrended cross-correlations analysis. Nonlinear Dyn..

[53] Mancini-Griffoli T., Ranaldo A. (2011). Limits to Arbitrage during the Crisis: Funding Liquidity Constraints and Covered Interest Parity.

[54] Chen K.S., Chen C.M., Lee C.C. (2017). Arbitrage, Covered Interest Parity and Cointegration Analysis on the NTD/USD Forex Market Revisited. Int. J. Econ. Financ. Issues.

[55] Wade R. (1998). The Asian debt-and-development crisis of 1997-?: Causes and consequences. World Dev..

[56] Chiodo A.J., Owyang M.T. (2002). A case study of a currency crisis: The Russian default of 1998. Fed. Reserve Bank St. Louis Rev..

[57] Bebczuk R., Galindo A. (2008). Financial crisis and sectoral diversification of Argentine banks, 1999–2004. Appl. Financ. Econ..

[58] Imbs J. (2010). The first global recession in decades. IMF Econ. Rev..

[59] Goodnight G.T., Green S. (2010). Rhetoric, risk, and markets: The dot-com bubble. Q. J. Speech.

[60] Luchtenberg K.F., Vu Q.V. (2015). The 2008 financial crisis: Stock market contagion and its determinants. Res. Int. Bus. Financ..

[61] Lane P.R. (2012). The European sovereign debt crisis. J. Econ. Perspect..

